# Perforación de intestino delgado secundaria a infección por citomegalovirus en un paciente con sida

**DOI:** 10.7705/biomedica.7439

**Published:** 2026-04-28

**Authors:** Laurent Marcela Osorio, María José Herrera, Brayan Leonardo Bayona, Julio César Insignares, Diego Viasus

**Affiliations:** 1 Facultad de Medicina, Universidad del Norte, Barranquilla, Colombia Universidad del Norte Facultad de Medicina Universidad del Norte Barranquilla Colombia; 2 Departamento de Patología, Hospital Universidad del Norte, Barranquilla, Colombia Hospital Universidad del Norte Departamento de Patología Hospital Universidad del Norte Barranquilla Colombia; 3 Departamento de Medicina Interna, Hospital Universidad del Norte, Barranquilla, Colombia Hospital Universidad del Norte Departamento de Medicina Interna Hospital Universidad del Norte Barranquilla Colombia

**Keywords:** citomegalovirus, perforación intestinal, íleon, huésped inmunocomprometido, síndrome de inmunodeficiencia adquirida, HIV., Cytomegalovirus, intestinal perforation, ileum, immunocompromised host, acquired immunodeficiency syndrome, HIV.

## Abstract

El citomegalovirus puede causar enfermedad diseminada o localizada en personas con HIV e inmunosupresión avanzada, quienes, por lo general, son aquellos con un número de linfocitos T CD4+ menor de 50 células/mm^3^ que no reciben, no cumplen o no responden a la terapia antirretroviral.

Este artículo reporta el caso de un paciente con HIV sin terapia antirretroviral, que fue admitido al servicio de urgencias por dolor abdominal. Durante la laparotomía, se encontró perforación del íleon, que requirió hemicolectomía derecha, anastomosis ileotransversa y drenaje por peritonitis fecaloide. En el estudio de histopatología, se documentaron múltiples inclusiones citomegálicas en células del estroma, epiteliales y endoteliales, con predomino en el íleon, en la zona de la perforación. Aunque la enfermedad por citomegalovirus del aparato digestivo es una de las complicaciones más comunes del sida, la perforación del íleon es una manifestación inusual con gran mortalidad.

## Abstract (en)

Cytomegalovirus can cause disseminated or localized disease in people living with HIV with advanced immunosuppression, usually those with CD4+ T-lymphocyte cell counts <50 cells/ mm^3^ who do not receive, complies, or does not respond to antiretroviral therapy.

We report on the case of a patient living with HIV without antiretroviral therapy who was admitted to the emergency department for abdominal pain. During laparotomy, perforation of the ileum was found, requiring right hemicolectomy, ileotransverse anastomosis, and drainage due to fecaloid peritonitis. The report of histopathology documented multiple cytomegalic inclusions in stromal, epithelial, and endothelial cells, predominantly in the ileum, at the level of the perforation area. Although cytomegalovirus disease in the gastrointestinal tract is one of the most common complications of HIV, ileal perforation is an unusual manifestation with high mortality.

El virus de la inmunodeficiencia humana (HIV, *Human Immunodeficiency Virus*) afecta a cerca de 38,4 millones de personas a nivel global. La región del Caribe presenta la segunda tasa de prevalencia más alta después de la región subsahariana de África ^1^^,^^2^. Las personas con HIV que no reciben tratamiento antirretroviral con inmunosupresión avanzada desarrollan con frecuencia infecciones oportunistas, entre ellas, la enfermedad por citomegalovirus, la criptococosis o la toxoplasmosis cerebral.

El citomegalovirus genera una variedad de síndromes clínicos en el paciente inmunocomprometido, entre los que se encuentran: síndromes febriles, hepatitis, neumonitis, retinitis, encefalitis y afectación del sistema digestivo ^3^. En las vías digestivas, los segmentos más afectados, en orden de frecuencia, son: el colon (47 %), el duodeno (21,7 %), el estómago (17,4 %), el esófago (8,7 %) y, raramente, el intestino delgado (4,3 %) ^4^.

El diagnóstico de la enfermedad por citomegalovirus (CMV), por lo general, se hace con base en la presentación clínica y, cuando es posible, por la presencia del virus en el tejido. En el examen de histopatología, se evidencian las inclusiones intranucleares e intracitoplasmáticas con la tinción de hematoxilina y eosina.

Ocasionalmente, el diagnóstico puede no ser claro; en estos casos, la prueba de la reacción en cadena de la polimerasa (PCR) para citomegalovirus en el humor vítreo o acuoso o en el líquido cefalorraquídeo puede ser útil para establecer el diagnóstico en la enfermedad ocular o del sistema nervioso central.

La presencia de anticuerpos séricos contra el citomegalovirus no establece el diagnóstico de la enfermedad por este virus. Sin embargo, un valor negativo de anticuerpos de inmunoglobulina G indica que es poco probable que el citomegalovirus sea la causa del proceso de la enfermedad.

La sospecha temprana de la coinfección de HIV y CMV y el diagnóstico de la enfermedad son cruciales. Se ha planteado, incluso, que el citomegalovirus podría contribuir a la persistencia de la infección por HIV mediante la inducción de células diana del HIV por medio de la codificación de un homólogo de la citocina IL-10 que produce un efecto inhibitorio de la respuesta inmunitaria del huésped ^5^^-^^7^.

A continuación, se presenta el caso de un hombre joven con síndrome de inmunodeficiencia adquirida que presentó dolor abdominal por perforación de íleon. El estudio histopatológico evidenció múltiples inclusiones citomegálicas en la zona de perforación, indicativas de enfermedad por citomegalovirus. Este caso adquiere importancia puesto que la afectación del íleon secundaria a infección por citomegalovirus es una condición poco común con un gran riesgo de mortalidad.

## Presentación del caso

Un hombre de 25 años, con infección por HIV diagnosticada ocho meses antes, consultó al servicio de urgencias por dolor abdominal y fiebre de dos días de evolución. También, presentaba un cuadro de seis meses de evolución de hematoquecia, diarrea y dolor abdominal en mesogastrio, de forma intermitente.

Como antecedentes, refirió asma y úlceras esofagogástricas. Recibía tratamiento con trimetoprim-sulfametoxazol y fluconazol. El paciente negó haber recibido en algún momento tratamiento antirretroviral.

En el examen físico presentaba signos de reacción inflamatoria sistémica y de irritación peritoneal. Se le practicaron exámenes de laboratorio y se evidenció aumento de la proteína C reactiva y neutrofilia sin leucocitosis (cuadro 1). También, se documentó una carga viral de HIV de 218 890 copias/ml con un número de linfocitos T-CD4 de 14/mm^3^.

**Cuadro 1.** Exámenes de laboratorio

Al ingreso al servicio de urgencias	Resultado
Proteína C reactiva (mg/l)	78,71
Nitrógeno úrico sérico (mg/dl)	21,80
Urea (mg/dl)	46,65
Creatinina sérica (mg/dl)	1,05
Glucemia (mg/dl)	94
Hemoglobina (g/dl)	13,4
Hematocrito (%)	39,3
Volumen corpuscular medio (fl)	77,8
Hemoglobina corpuscular media (pg)	26,5
Ancho de distribución plaquetario (%)	14,7
Plaquetas (por µl)	418 000
Leucocitos (por µl)	8760
Neutrófilos (por µl)	8020
Linfocitos (por µl)	470
Durante la hospitalización	
Anticuerpos de superficie de hepatitis B	No reactivo
VDRL	No reactivo
VDRL Anticuerpos contra la hepatitis C	Negativo
Anticuerpos contra la proteína *core* de la hepatitis b	Negativo
Cultivo de líquido peritoneal	*E. coli*
Coprocultivo (*Shigella* spp. y *Salmonella* spp.)	Negativo
Tinción de Ziehl-Neelsen en heces	Negativo
Antígeno sérico para criptococo	Negativo
PCR y tinción de Ziehl-Neelsen para *M. tuberculosis* en líquido peritoneal	Negativo
Antígeno para *G. lamblia, E. hystolitica* y toxina de *Clostridium* spp.	Negativos

En la ecografía abdominal, se encontraron un proceso inflamatorio cecal y líquido libre en el espacio hepatorrenal. El paciente fue sometido a laparotomía, durante la cual se evidenció una perforación intestinal en el íleon a 1 m de la válvula ileocecal y peritonitis fecaloide. Se practicó una hemicolectomía derecha, con anastomosis ileotransversa y drenaje de la peritonitis.

El paciente recibió tratamiento con ceftriaxona y metronidazol intravenoso. En los cultivos del líquido peritoneal, se aisló *Escherichia coli* con fenotipo de resistencia a inhibidores (2br). Además, se le practicaron los siguientes exámenes de laboratorio (cuadro 1), todos con resultados negativos:

- antígeno sérico para criptococos,
- antígenos en heces para *Giardia lambia y Entamoeba histolytica*,
- tinción de Ziehl-Neelsen en heces,
- reacción en cadena de la polimerasa para *Mycobacterium tuberculosis* en líquido peritoneal, y
- toxinas A y B para *Clostridioides difficile.*

En el periodo posoperatorio, presentó una adecuada evolución con resolución de la sintomatología, por lo que se le dio egreso con indicación de control ambulatorio una vez obtenido el resultado del estudio histopatológico.

Posteriormente, requirió reingreso hospitalario por recurrencia del dolor abdominal y ausencia de evacuaciones. Se le practicó una nueva laparotomía exploratoria, en la que se descartó una nueva perforación y se liberaron adherencias.

**Figura 1.**
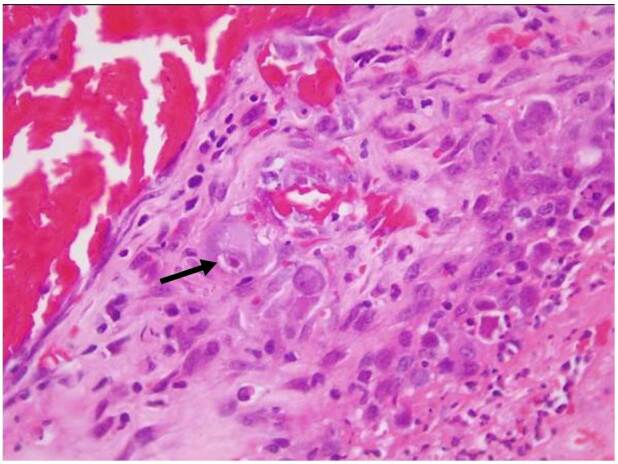
Se observan cambios citopáticos sugestivos de infección por citomegalovirus Íleon: agrandamiento celular y halo perinuclear con aspecto de ojo de búho, hematoxilina y eosina, 40X

En el reporte de histopatología, se documentó necrosis transmural, hemorragia y formación de absceso en el íleon. Además, se identificaron focos de necrosis de mucosa y de formación de absceso mural de colon. Se evidenciaron múltiples inclusiones citomegálicas en células del estroma, epiteliales y endoteliales, con predominio en el íleon en la zona de perforación (figura 1). Por lo anterior, se le inició tratamiento con ganciclovir intravenoso. El paciente presentó buena evolución y se le dio egreso con seguimiento ambulatorio.

## Consideraciones éticas

El paciente autorizó la comunicación de su caso mediante un consentimiento informado.

## Discusión

Se describe el caso clínico de un paciente con diagnóstico de HIV e inmunosupresión avanzada, que presentó dolor de abdomen agudo secundario a una perforación del intestino delgado. El análisis histopatológico fue concluyente para infección por citomegalovirus y el paciente presentó una respuesta satisfactoria al tratamiento con ganciclovir.

El citomegalovirus es un virus herpes que infecta al 60 % de los adultos en los países desarrollados y a más del 90 % en los países en desarrollo ^8^. Este virus puede causar enfermedad diseminada o localizada en personas con HIV e inmunosupresión avanzada. Sin embargo, la incidencia de nuevos casos de enfermedad de órgano blanco por citomegalovirus ha disminuido en el 95 % o más con el advenimiento de la terapia antirretroviral.

La retinitis es la manifestación clínica más común de la enfermedad de órgano blanco por citomegalovirus en personas con HIV. Otras manifestaciones clínicas incluyen: colitis, neumonitis, esofagitis y enfermedad del sistema nervioso central o periférico.

Las manifestaciones gastrointestinales ocupan cerca del 10 % de los trastornos por citomegalovirus entre los casos de sida ^5^. Clínicamente, estas manifestaciones se presentan según la región afectada. En el esófago, se presenta odinofagia y fiebre; en el estómago, dolor subesternal y epigástrico; en el intestino delgado, dolor abdominal generalizado y diarrea, y en el colon, anorexia, pérdida de peso y diarrea ^9^. Cabe destacar que la enfermedad por citomegalovirus suele ser multifocal, como en el caso reportado ^4^^,^^10^.

Se debe hacer seguimiento de los pacientes con inmunosupresión grave para detectar de forma temprana la enfermedad por citomegalovirus. Así, se podría indicar la necesidad de ordenar estudios complementarios para el inicio oportuno del tratamiento con el fin de evitar complicaciones que conllevan gran morbilidad y mortalidad.

El diagnóstico de la enfermedad por citomegalovirus se hace, generalmente, con base en la presentación clínica y, cuando es posible, por la presencia del virus en el tejido. La sospecha de la enfermedad gastrointestinal debe basarse en los síntomas, la visualización de lesiones por vía endoscópica y la presencia de inclusiones de tipo A de Cowdry ^11^. El diagnóstico puede confirmarse mediante inmunohistoquímica, la cual tiene sensibilidad y especificidad del 97 y del 100 %, respectivamente ^11^.

Esta confirmación es necesaria debido a que, ni la detección viral mediante análisis de sangre ni la serología en un paciente inmunocomprometido, pueden confirmar la presencia de la enfermedad en un órgano diana ^9^. El cultivo de citomegalovirus o la detección de ADN de este virus mediante PCR a partir de una biopsia tampoco son suficientes para establecer el diagnóstico de colitis o esofagitis por citomegalovirus en ausencia de cambios histopatológicos, porque los pacientes con disminución de los linfocitos T-CD4 pueden eliminar el citomegalovirus y tener cultivos positivos en ausencia de enfermedad clínica.

Uno de los factores genéticos determinantes del tropismo del citomegalovirus por células epiteliales y mesenquimatosas, se encuentra en el locus *UL131A/UL130/UL128* del genoma del virus ^5^. Los productos del gen *UL131A-UL128* se unen a las glucoproteínas virales H y L, dando como resultado el complejo viral que le permite al virus entrar en las células del huésped ^12^^,^^13^.

El citomegalovirus es capaz de establecer latencia en células endoteliales y en células progenitoras hematopoyéticas (CD34) para, posteriormente, reactivarse y reinfectar el epitelio ^6^^,^^14^.

El enfoque terapéutico de la enfermedad por citomegalovirus debe individualizarse en función de la tolerancia a los medicamentos sistémicos, la exposición previa a los fármacos contra citomegalovirus y, posiblemente, la ubicación de las lesiones ^3^^,^^13^.

Para los pacientes que tienen compromiso gastrointestinal, muchos especialistas en HIV recomiendan la terapia durante 21 a 42 días o hasta que los signos y síntomas hayan desaparecido. El ganciclovir es, en general, el tratamiento de elección y puede cambiarse a valganciclovir oral una vez que el paciente pueda tolerar y absorber los medicamentos orales. El foscarnet puede utilizarse como alternativa si hay toxicidad relacionada con el ganciclovir o en casos de virus resistentes al ganciclovir. El valganciclovir oral se puede utilizar en pacientes con enfermedad leve.

## Conclusión

La detección de la enfermedad gastrointestinal por citomegalovirus es esencial en los pacientes con HIV e inmunosupresión avanzada. Los signos y síntomas de esta enfermedad pueden variar según la región afectada del tubo digestivo. Por lo tanto, es crucial considerar esta infección como una posible causa en los pacientes con síntomas gastrointestinales, una vez descartadas otras causas etiológicas.

Este caso resalta la importancia de correlacionar los síntomas clínicos, los hallazgos de imágenes diagnósticas y los resultados del estudio histopatológico para confirmar el diagnóstico. El establecer el tratamiento de forma inmediata es fundamental; de lo contrario, las complicaciones subsiguientes pueden conducir a falla multiorgánica y muerte.
